# New insights into the autofluorescence properties of cellulose/nanocellulose

**DOI:** 10.1038/s41598-020-78480-2

**Published:** 2020-12-07

**Authors:** Qijun Ding, Wenjia Han, Xia Li, Yifei Jiang, Chuanshan Zhao

**Affiliations:** State Key Laboratory of Biobased Material and Green Papermaking, Qilu University of Technology, Shandong Academy of Sciences, Jinan, 250353 China

**Keywords:** Chemistry, Materials science, Nanoscience and technology, Optics and photonics

## Abstract

This work explored the fluorescence properties of nano/cellulose isolated from bleached softwood kraft pulp by TEMPO oxidation. Fluorescence spectra showed that all samples exhibited a typical emission peak at 574 nm due to the probabilistic formation of unsaturated bonds by glycosidic bonds independent of lignin. Increasing the excitation wavelengths (510–530 nm) caused red shift of fluorescence emission peaks (570–585 nm) with unchanged fluorescence intensity. Conversely, changing acid/alkaline conditions led to an increase of fluorescence intensity with no shifting of fluorescence emission peak. This can be attributed to an increase in the polarity of the solution environment but does not cause interaction of functional groups within the system identified by generalized two-dimensional correlation fluorescence spectroscopy. This study provides new insight in applying nano/cellulose with special luminous characteristics in biomedicine area such as multi-color biological imaging and chemical sensing.

## Introduction

Cellulose, as the main skeletal component in plants, is an inexhaustible polysaccharide-based raw material with favorable structure and properties^1, 2^. It is essentially composed of homopolymerized β-D-glucopyranose units with a unique structural grade in its biological origin. However, its properties, functionality, durability and uniformity should not be stayed at traditional cellulosic materials, but need to be developed for the next generation of cellulose based products^3, 4^. Once cellulose extracted in a nanoscale, most of the defects associated with the hierarchical structure would be removed. The new construction of cellulose-based “building blocks” can be used for the next generation of cellulose-based composites.

Extensive research had been focused on the methods for isolating cellulose nanocrystals (CNCs)/cellulose nanofibrils (CNF) and investigating their functional modifications. Based on their intriguing properties such as low cost and low toxicity, optical clarity, reproducibility, biodegradability, low thermal expansion, nanocellulose’s application area could be extensively expanded when it was functionalized^3, 5^. The most important features of nanocellulose for structure applications include size, strength/toughness, modulus, degree of polymerization, and surface function^6, 7^. Although the structure and surface functionalization of nanocellulose had been applied to improve the structural and mechanical properties of composites, its relationship to fluorescence properties have rarely been reported, which may inspire a more extensive application^8^. Autofluorescence means that under a certain wavelength of excitation light, electrons in a substance can enter an excited state and then transition back to the ground state to produce a certain wavelength of light. In past studies, Castellan et al. believe that the fluorescence of pulp is only attributed to lignin, which may be phenylcoumarin and coniferyl structure^9^. However, Olmstead and Gray^10^ reported the fluorescence properties of mechanical pulp sheets. They concluded that cellulose showed a relatively high characteristic emission regardless of its source. And removal of almost all of the lignin from both pulps by acidic chlorite treatment did not significantly reduce fluorescence. They concluded that the fluorescent properties of the pulp are not overly dependent on the formal lignin-cellulose bonds present in the mechanical pulp: instead, lignin and cellulose act independently^11^. Therefore, the issue of cellulose fluorescence properties remains controversial. At present, the fluorescence properties of nanocellulose had rarely been reported, as well as its elaborate characterization, intensity regulation and application. Kalita et al^12, 13^. used saw dust and rice husks to prepare nanocellulose and found its fluorescence properties, but the mechanism of fluorescence was not further explored.

In this work, we reported on the fluorescence properties of cellulose nanofibrils (CNFs) isolated from bleaching chemical pulp using typical TEMPO-mediated oxidation. Regarding the characterization of CNFs in various states, a series of modern characterization methods were used to expound the relationship between structural changes and fluorescence properties concerning their degree of polymerization, chemical structures, microstructures, chemical component for better comprehension of the fluorescence mechanisms. The effect of changes in the external environment (excitation wavelength and pH) on fluorescence properties was further investigated. This will be beneficial for the development and application of cellulose-based fluorescent materials.

## Results and discussion

### Characterization of nano/cellulose

CNFs are extracted from BSKP by TEMPO oxidation system, followed by homogenized. The chemical composition of BSKP and derived CNFs were chemically analyzed and were represented in Table 1. The native cellulose content of BSKP estimated at 76.3% were enhanced to about 90% in the derived CNFs samples. The lignin and hemicellulose content of BSKP were substantially lowered following the oxidation treatment of the raw fibers, resulting in the breakdown of the lignocellulosic structure and improved defibrillation^14^. It could also be seen that the carboxyl content of the surface of the nanocellulose gradually increased with the degree of oxidation.

**Table 1:** Properties comparison and component analysis of CNFs and BSKP.

Samples	DP	Cellulose (%)	Lignin (%)	Hemicellulose (%)	Carboxyl content (mmol/g)
BSKP	874.3 ± 5.2	76.23	3.94	15.83	Nil
CNF-1	325.4 ± 3.8	89.62	Nil	3.52	0.41
CNF-2	312 ± 6.4	87.23	Nil	2.14	0.79
CNF-3	294 ± 2.8	89.52	Nil	1.72	1.18

**Figure 1:**
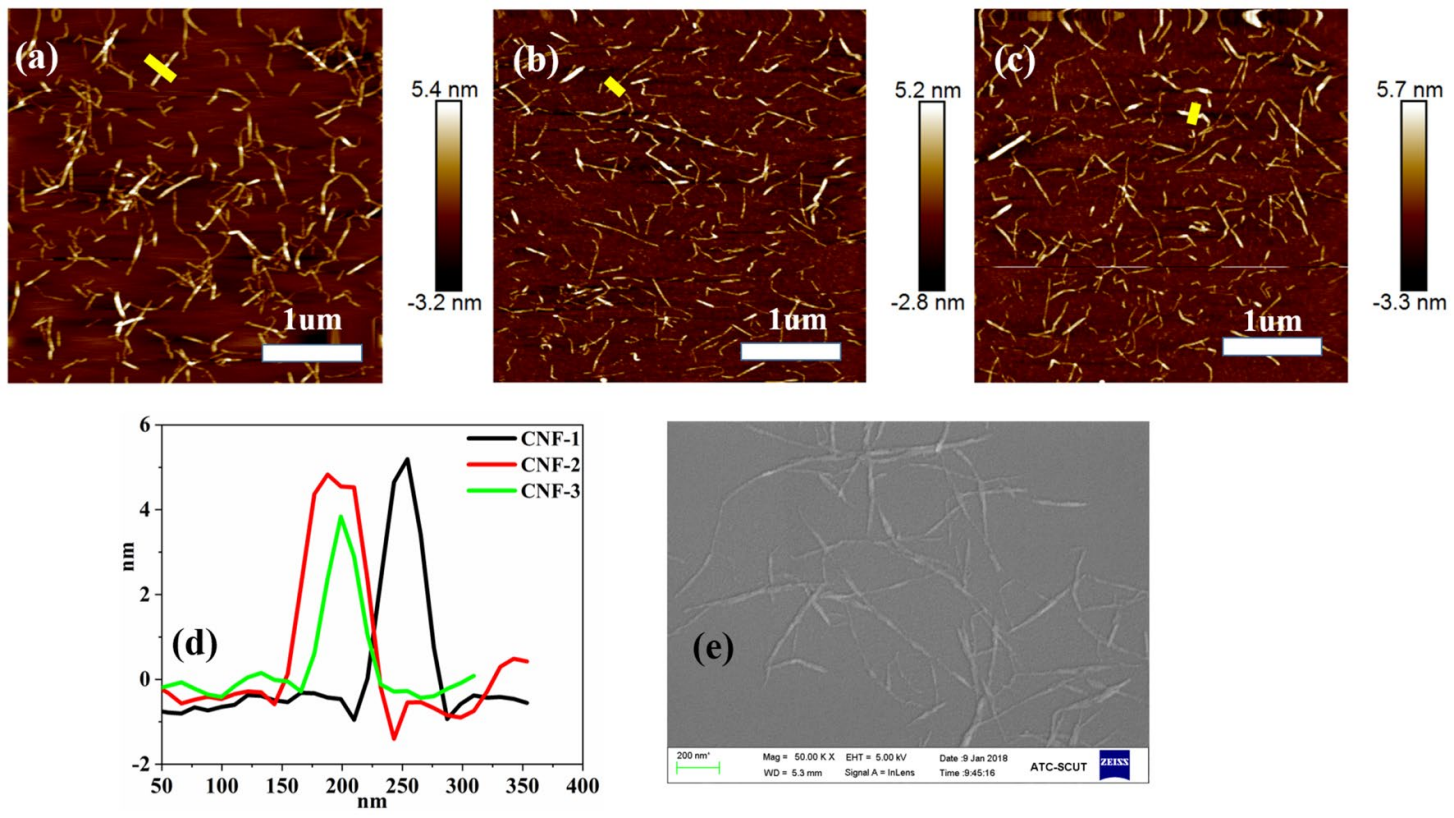
AFM topography images showing height measurements of (**a**) CNF-1, (**b**) CNF-2, (**c**) CNF-3. (**d**) AFM line scan of CNFs image. The height data corresponds to the yellow bar in the AFM images. (**e**) SEM image of CNF-3.

Figure 1 showed that the AFM topography of CNFs were representative image of the morphology and phase of the extracted nanofibers, in the ScanAsyst mode. The high-pressure homogenized treatment followed by chemical treatments resulted in the defibrillation of the cellulose nanofibers, evident in the AFM images revealing the separation of these nanofibers. It could be observed from the AFM pictographs that all CNFs had a relatively constant cross-section and had kinks and sharp bens between straight segments. The average length and diameter of the CNFs, calculated from the electron micrographs were found to be in the range of 300–800 nm and 2–10 nm, respectively.

### Fluorescence properties of nano/cellulose

**Figure 2:**
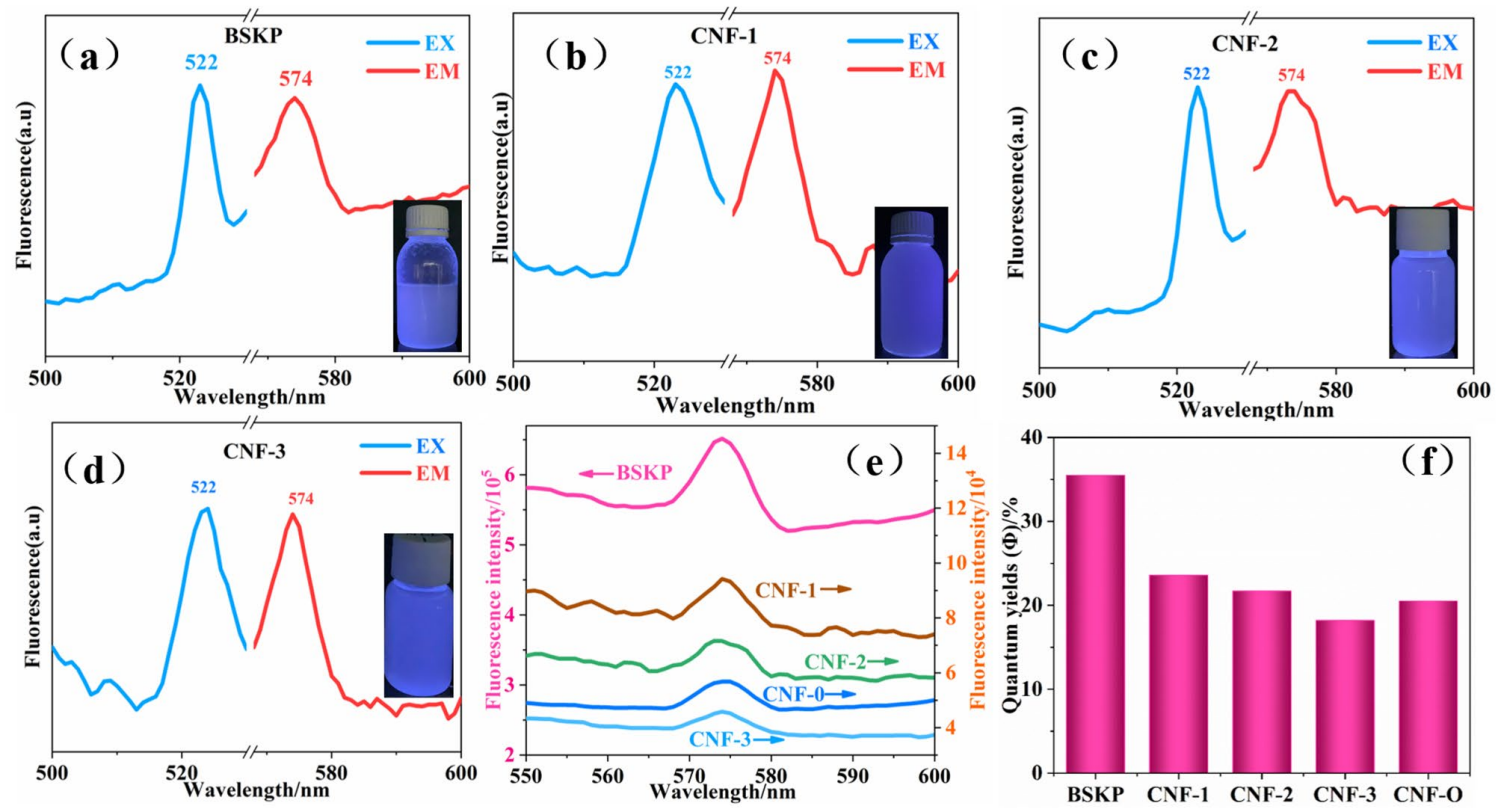
Fluorescence spectrum with inset showing the fluorescence pictographs (EX: excitation spectrum, EM: emission spectrum) of (**a**) BSKP, (**b**) CNF-1, (**c**) CNF-2, (**d**) CNF-3. (**e**), (**f**) Comparison of fluorescence intensity and QY.

For the assessment of the optical properties, the fluorescence spectra of the aqueous dispersions of BSKP and CNFs were recorded (Fig. 2). The excitation and emission spectra of BSKP and CNFs exhibited the optimal absorption at 522 nm and 574 nm, respectively. The inset showed the pictographs of BSKP and CNFs dispersed in water, under UV-illumination. The samples appeared colorless under visible light but demonstrated a bright blue fluorescence under UV illumination. It is thus proved that nanocellulose is a photoluminescent material with fluorescence properties. Compared to the raw material, the fluorescence intensities of CNFs were clearly decreased and further decreased as the degree of oxidation increased (Fig. 2e). The quantum yields (QY) for the CNFs were calculated from the emission spectra using rhodamine B (emission range 550–600 nm) as a reference. As shown in Fig. 2f, the QY of BSKP, CNF-0, CNF-1, CNF-2 and CNF-3 were 35.5%, 20.5%, 23.6%, 21.7% and 18.2%, respectively. One of the reasons for the decrease in fluorescence intensity and QY may be due to the introduction of carboxyl groups which acted as an electron acceptor which weakened the flow of π electrons in the system and then limited the fluorescence property^15–17^.

### Mechanism of fluorescence properties of nano/cellulose

**Figure 3:**
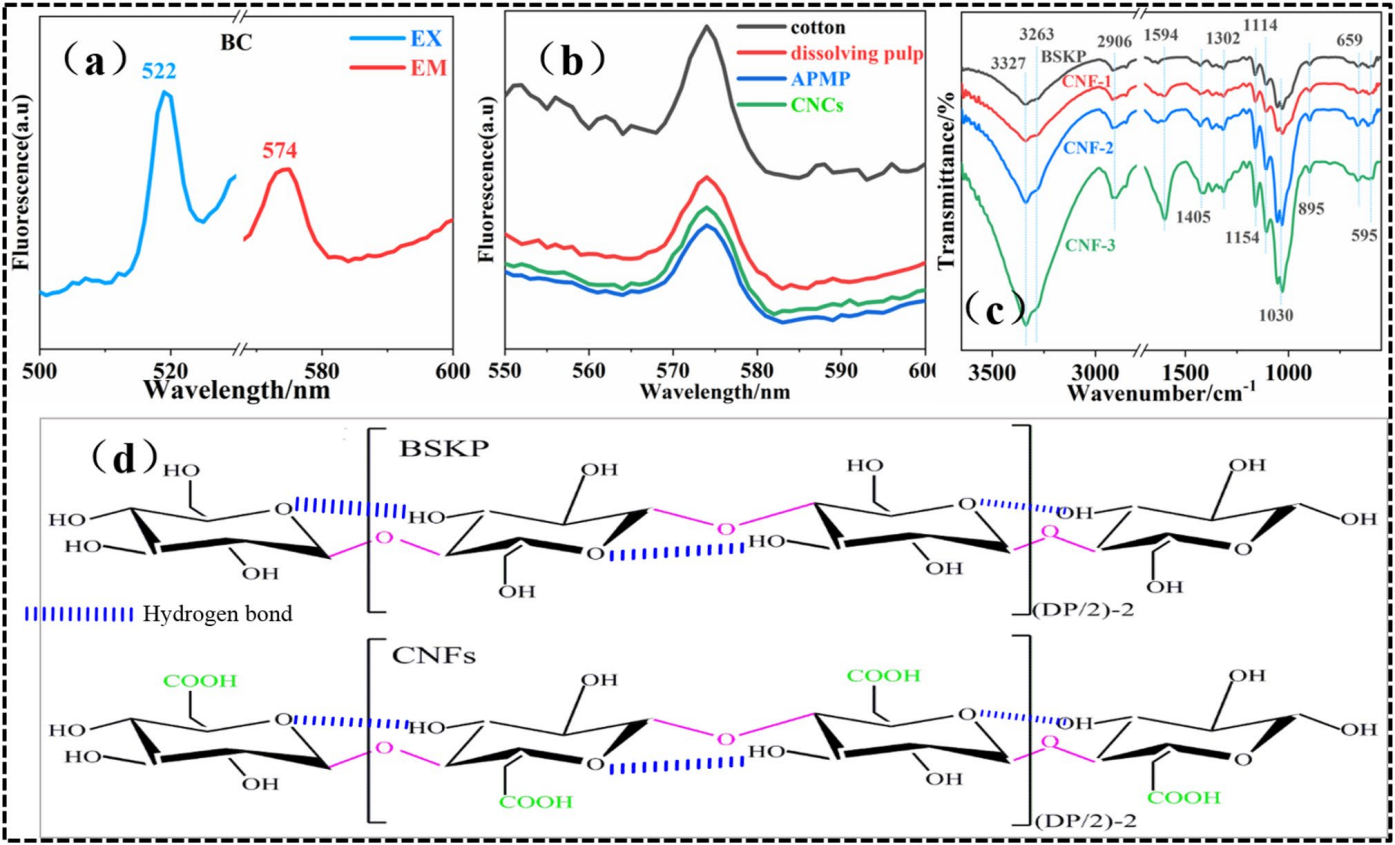
(**a**) Fluorescence spectrum of BC. (**b**) Comparison of fluorescence properties of cotton, dissolving pulp, APMP, CNCs. (**c**) Comparison of FTIR spectra of BSKP, CNF-1, CNF-2 and CNF-3. (**d**) Schematic diagram of the molecular chain structure of BSKP and CNFs.

It is well known that lignin has natural fluorescence properties^18, 19^. In order to verify that the fluorescence properties of nano/cellulose in the wavelength range of 500–600 nm were independent of lignin, the fluorescence spectrum of high purity (Cellulose content ≥ 99.9%) BC was investigated, as shown in Fig. 3a. The excitation and emission spectra of BC showed the optimal absorption peaks at 522 nm and 574 nm, respectively. And the cotton, dissolving pulp, APMP, and CNCs all showed significant emission peaks at 574 nm (Fig. 3b). It can be concluded that the fluorescence properties of nano/cellulose (range of 500–600 nm) are caused by its own structure and are independent of the presence of lignin.

There are two necessary conditions for a substance to produce fluorescence: (1) The energy absorbed by a photon undergoing multiple invariant transitions is less than the energy required to break its weakest chemical bond. (2) A fluorophore containing an unsaturated bond should be included in the substance^20^. The FTIR spectra of BSKP and CNFs were shown in Fig. 3c. The broad region 3600–3200 cm^−1^ was related to –OH vibrations. The wide peak can be assigned to three types of O(2)H–O(6) intramolecular, O(3)H–O(5) intramolecular, and O(6)H–O(3) intermolecular hydrogen bonding^21^. The peak observed at 2906 cm^−1^ was due to the aliphatic saturated –CH_2_ and –CH_2_OH stretching vibration of polysaccharides^22^. The small absorption peak at the 1594 cm^−1^ band was designated as the bound water and carboxylate from the bleaching process. As previously published reports, the acetyl and uronic ester groups of the celluloses were represented by the characteristic peak at 1405–1302 cm^−1^^23^. Absorption band at 1030 cm^−1^, the strongest band across the cellulose spectra, was assigned to CO stretching at the C3 position. The absorption peak of 670–550 cm^−1^ was related to CH deformation and OH out-of-plane bending^24^. The minor signature at 895 cm^−1^ was attributed to the β-glycosidic linkages of glucose ring of cellulose. The presence of β-glycosidic bond of hemiacetal structure may be responsible for the fluorescence property as shown in Fig. 3d. It seems that the random movement of free electrons in the cellulose molecular chain may probabilistically form unsaturated double bonds with glycosidic bonds.

**Figure 4:**
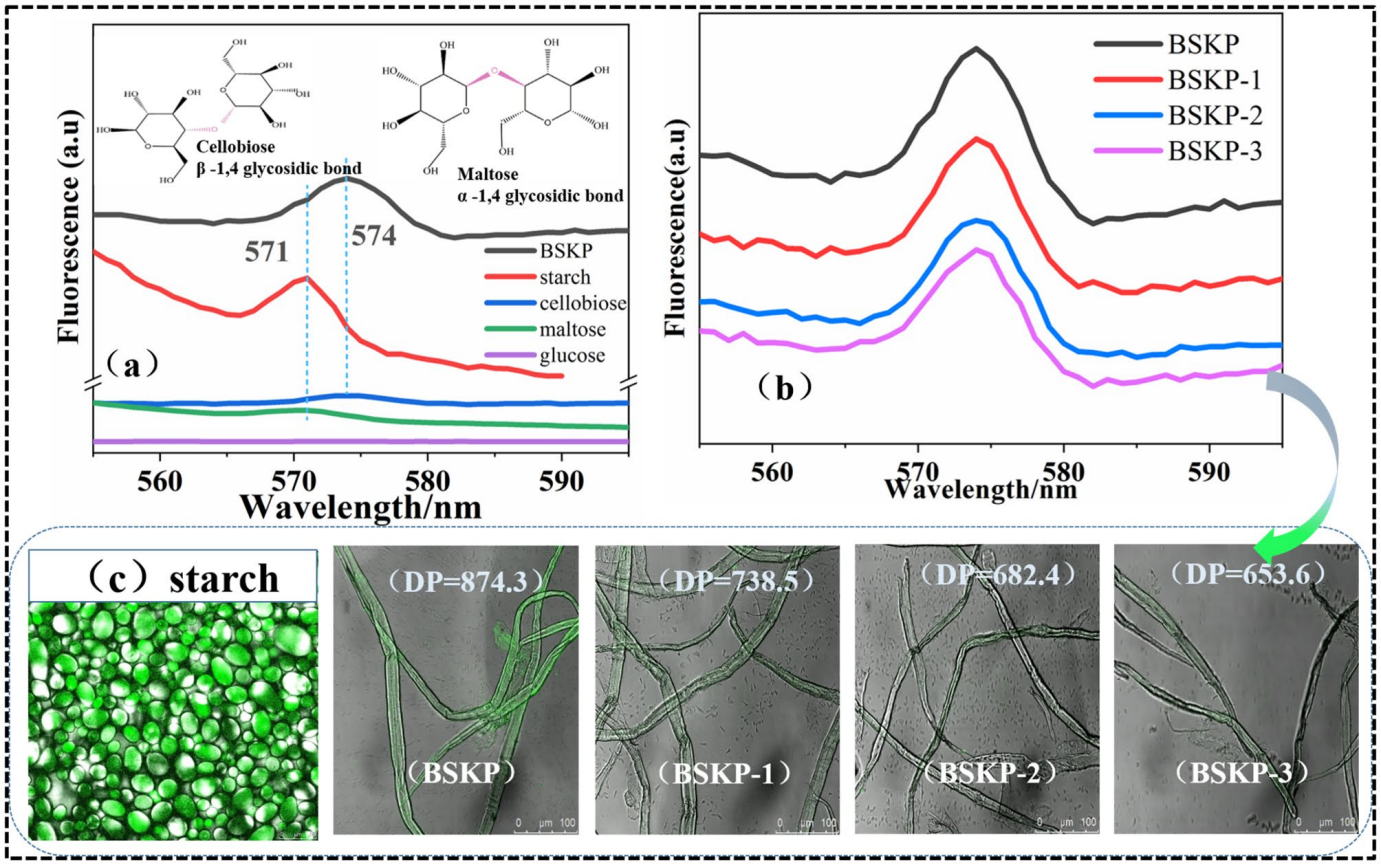
Comprehensive comparison of the fluorescent properties caused by glycosidic bonds. (**a**) Comparison of fluorescence properties of α-1, 4 glycosidic bond and β-1, 4 glycosidic bond. Inset: molecular configuration of α-1, 4 glycosidic bond and β-1, 4 glycosidic bond. (**b**) Comparison of fluorescence properties of BSKP, BSKP-1, BSKP-2 and BSKP-3. (**c**) LSCM images of BSKP, BSKP-1, BSKP-2 and BSKP-3 and starch.

The glycosidic bond is a specific type of chemical bond connecting sugar group and sugar group including β-1, 4 glycosidic bond and α-1, 4 glycosidic bond, which are mainly present in cellulose and starch, respectively. Cellulose and starch contain the same basic building blocks (glucose) and different repeating units (cellobiose and maltose) as shown in Fig. 4a. As could be seen from Fig. 4a, maltose and starch showed a distinct emission peak at 571 nm. Cellobiose and cellulose exhibited a distinct emission peak at 574 nm. The glucose solution showed no emission peaks at 571 nm and 574 nm. It can be concluded that the fluorescence properties of cellulose and starch in the range of 550–600 nm are mainly caused by the probability that glycosidic bonds between glucose molecules become double bonds. The change in the position of the emission peak of cellulose and starch can be attributed to the difference in the configuration of the cellobiose and maltose molecules as shown in Fig. 4a, inset.

Figure 4b showed the relationship between the number of cellulose glycosidic bonds (degree of polymerization) and the fluorescence intensity. The DP of BSKP, BSKP-1, BSKP-2 and BSKP-3 were 874.3, 738.5, 682.4 and 653.6, respectively. As the DP of cellulose decreased, the fluorescence intensity gradually decreased. The fluorescence signal of the LSCM images was also gradually weakened (Fig. 4c). It can be concluded that the DP is an important factor affecting the fluorescence properties of natural cellulose that has not been chemically modified. A significant decrease in the fluorescence intensity of CNFs is attributable to the introduction of π-conjugated groups (–COOH) relative to BSKP^17^. The presence of a more polar carboxyl group may reduce the probability that the glycosidic bond will adsorb electrons to become an unsaturated bond.

### Effect of external environment on fluorescence properties

**Figure 5:**
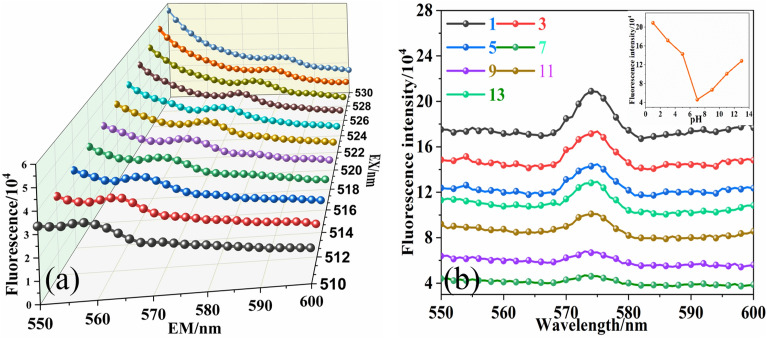
Effect of the external environment on the fluorescent properties of CNF-3. (**a**) Excitation wavelength, (**b**) different pH. Inset: The trend of the change in fluorescence intensity at different pH.

As an emerging nanomaterial, nanocellulose is similar to other fluorescent materials in that its fluorescent properties are susceptible to the external environment. In Fig. 5a, the emission peaks of CNF-3 showed a significant red shift (570–585 nm) as increasing the excitation wavelength (510–530 nm). And the fluorescence intensity did not change. This excitation-dependent behavior can be attributed to the the presence of a hemiacetal structure (Fig. 3c)^25^. These defects produced by unsaturated groups as capture centers of excitons give rise to the surface-state-related fluorescence^26^. They can modulate the emission wavelength at different excitation wavelengths, which is important for some practical applications, such as photoluminescent material.

The pH of the system was one of the important factors affecting the luminescent properties of fluorescent substances. The fluorescence spectra of nanocellulose at different pH was shown in Fig. 5b. CNF-3 exhibited excellent fluorescence properties regardless of acidic or basic conditions. And the position of the emission peak did not change. This property allows nanocellulose to be used in different environments. The fluorescence intensitiey of CNF-3 was increased with acid/basic enhancement.

**Figure 6:**
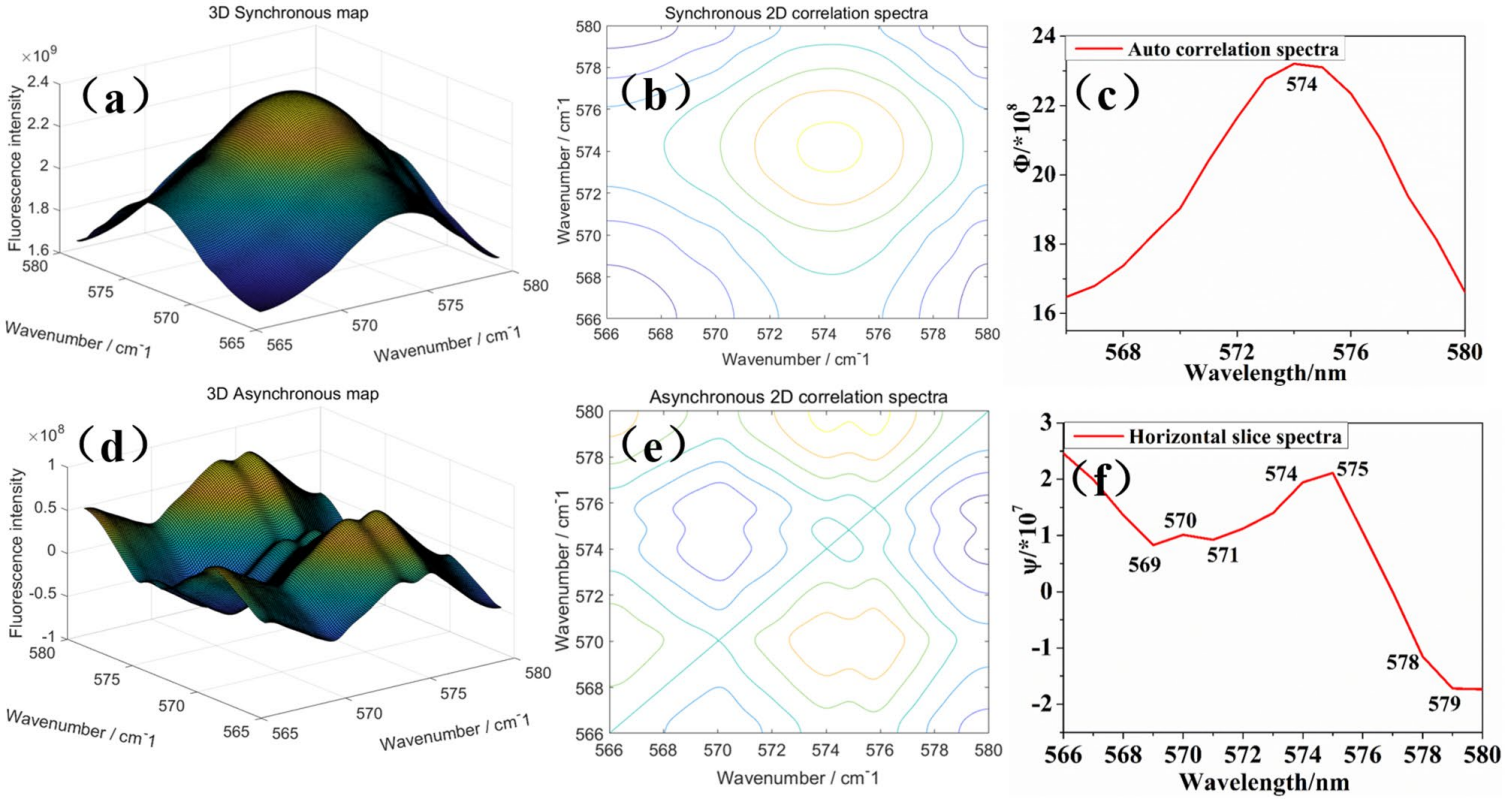
Two-dimensional correlation spectrum of CNF-3 at different pH. (**a**) 3D synchronous map. (**b**) Synchronous 2D correlation spectra. (**c**) Auto correlation spectra. (**d**) 3D asynchronous map. (**e**) Asynchronous 2D correlation spectra. (**f**) Horizontal slice spectra.

To further investigate the effects of different pH on the fluorescence properties of CNF-3, two-dimensional correlation fluorescence spectra (2DCOS) were applied. 2DCOS was considered to be an important characterization of the spectrum because it directly extends the spectral signal to two dimensions to improve spectral resolution and reveals the relationship and sequence of changes between groups^27–29^. 2DCOS of CNF-3 with pH as external disturbance factors were shown in Fig. 6. The 3D synchronous map of CNF-3 exhibits a strong automatic peak at (574, 574) that were positive, which represented the sensitivity (increased or decreased simultaneously) of the emission peak to external disturbance factors (Fig. 6a–c). No significant cross peaks were observed indicating that there was no intermolecular interaction between the functional groups on the surface of the nanocellulose^30^. We could conclude that the increase in fluorescence intensity was due to the fact that the change in pH increased the polarity of the solution but did not alter the intramolecular structure of the nanocellulose. And the increase in the polarity of the suspension also facilitated the generation of fluorescence^31^.

The two-dimensional asynchronous correlation spectrum only had cross peaks outside the diagonal line and had no peaks on the diagonal line^32^. The horizontal slice spectra can be extracted from the asynchronous 2D correlation spectrum. As shown in Fig. 6d–f, the two-dimensional correlation spectrum of the CNF-3 exhibited significant cross peaks at 569, 570, 571, 574, 575, 578 and and 579 nm. This indicated that the reorientation behavior of the dipole transition moments of their respective fluorescence absorptions was independent. And the interaction of the functional groups of these absorption peaks is not “related”^29^.

## Conclusion

This work showed that nano/cellulose fluorescence came from the possibility that the glycosidic bonds in the system randomly form unsaturated bonds. The lower DP and the introduction of electron acceptors groups (–COOH) resulted in a significant decrease in the fluorescence intensity of nano/cellulose. The essential ingredient of cellulose and lignin has no significant effect on the fluorescence intensity of nanocellulose. Moreover, the fluorescence emission peak of CNF could undergo blue/red shift and high/low regulation with different excitation wavelengths and pH values. Besides, the sensitive wavenumber position of nanocellulose produce by external disturbance conditions and the interaction between each group were thoroughly studied by two-dimensional correlation fluorescence spectroscopy.

## Methods

### Material

The cellulose source of never-dried bleached softwood kraft pulp (BSKP), alkaline peroxide mechanical pulp (APMP) and dissolving pulp were provided by Tai Yang Company (Shandong, China). Cotton was purchased from Guangzhou Nayong Cosmetics Co., Ltd. CNCs are supplied by Northern Century Cellulose Materials Ltd. Sodium hypochlorite (NaClO, AR, 7.5%), sodium bromide (NaBr, AR, 99%), 2, 2, 6, 6-tetramethylpiperidine (TEMPO, purity ≥ 99.9%) and rhodamine B (Φ = 0.31) were purchased from Sigma-Aldrich. Commercially available CNF (CNF-0) and bacterial cellulose (BC, Cellulose content ≥ 99.9%.) was purchased from Hainan Yide Food Co., Ltd. Potato starch (CAS: 9005-25-8), cellobiose, maltose, glucose and reagents were purchased from Qianhui Chemical Reagent Co., Ltd. (Guangzhou, China) and were of analytical grade unless otherwise stated.

### Isolation of CNFs from BSKP

The preparation of CNFs was carried out according to the previously published acid hydrolysis method^14, 33–35^. In brief, CNFs were defibrillated via TEMPO oxidation employing 2.0/4.0/6.0 mmol/g NaClO/cellulose at pH 10.0. Adjustment of pH at 7.0 with 0.5 M of NaOH, followed by homogenization (Microfluidizer LM20, USA) at 25000PSI for 5 times. The samples were respectively referred to as CNF-1, CNF-2, CNF-3.

### Mechanically refined

The concentration of BSKP was adjusted to 1% and then ground by Super Masscolloider (MKCA6-2, Japan). The grinding cycle was performed at intervals of − 50 μm, − 80 μm, and − 100 μm for 10 times. Finally, three samples with different degrees of polymerization were obtained. The three samples obtained by grinding were respectively referred to as BSKP-1, BSKP-2 and BSKP-3.

### Chemical analysis

Chemical analysis of BSKP and CNFs were performed to estimate the total cellulose and lignin content present in the samples. The cellulose content in the samples was estimated based on the TAPPI standard T203 CM-09. The total lignin content present in the samples was determined according to the TAPPI standards T222 OM-02 and T222 OM-11. Component analysis of all samples was determined by a Dionex (Sunnyvale, CA) HPLC system (ICS-5000) equipped 165 with a GP40 gradient pump, an anion exchange column (CarboPac PA20 and a guard) 166 and an ED40 electrochemical detector according to previously reported methods^36^.

### Determination of viscosity average molecular weight

The degree of polymerization (DP) of the BSKP fibers and CNFs were determined by intrinsic viscosity measurement according to previously reported methods^37^. The viscosity of the fibers dissolved in the 0.5 M methylene diamine hydroxide solution at 25 ± 0.5 °C was estimated using an Ubbelohde viscometer in a water bath with a thermostat. The intrinsic viscosity [η] was obtained according to the ASTM method (ASTM D1795-94, 2001). DP was calculated by the formula (1)^38^:

1 $${\text{Dp}}^{0.905} = 0.75\left[\upeta \right]$$

### Characterization

The carboxyl groups content of CNFs were determined by conductivity titrator (ZDJ-5B-G, INESA Scientific InstrumentCo., Ltd). FTIR spectra were collected on a VERTEX 70 (Bruker, Germany) Fourier transform infrared spectrometer. The test specimens were prepared by a standard KBr pellet method. Spectra were recorded between 600 and 4000 cm^−1^ at a resolution of 4 cm^−1^, and 64 scans were collected for each spectrum. Fluorescence spectra of all samples and fluorescence spectra at different pH and excitation wavelengths were performed with a HORIBA Scientific FluoroMax-4 equipped with a 150 W xenon lamp and the quantum yields (Φ) were calculated. Emission spectrum (550–600 nm) was acquired at an excitation wavelength of 520 nm. Excitation spectrum (500–550 nm) was acquired at an emission wavelength of 570 nm. The widths of both the excitation slit and emission slit were set at 3.0. The quantum yields (Φ) of samples were carried out using rhodamine B as a reference having an emission range of 550–600 nm^39^. Fluorescent images of BSKP and potato starch were measured by Laser Scanning Confocal Microscopy (LSCM) equipped with 458 nm/488 nm/514 nm Ar lamps (Leica TCS-SP8, Germany). Excitation light at a wavelength of 514 nm is used to excite fiber fluorescence. MultiMode 8 SPM (Bruker, USA) atomic force microscope (AFM) was used to image the nanocellulose surface morphology. The further image processing was finished using Nanoscope Analysis software (version 1.5, Bruker Corporation). All generalized two-dimensional correlation spectral (2DCOS) analyses were performed in 2D-shige software^28, 40, 41^. Images of two-dimensional correlation spectra were implemented using a homemade Matlab program.
